# Fungal and oomycete pathogens and heavy metals: an inglorious couple in the environment

**DOI:** 10.1186/s43008-022-00092-4

**Published:** 2022-04-25

**Authors:** Joanna Gajewska, Jolanta Floryszak-Wieczorek, Ewa Sobieszczuk-Nowicka, Autar Mattoo, Magdalena Arasimowicz-Jelonek

**Affiliations:** 1 Department of Plant Ecophysiology, Faculty of Biology, Adam Mickiewicz University, Uniwersytetu Poznańskiego 6, 61-614 Poznań, Poland; 2 Department of Plant Physiology, Poznań University of Life Sciences, Wołyńska 35, 60-637 Poznań, Poland; 3 Department of Plant Physiology, Faculty of Biology, Poznań Adam Mickiewicz University, Uniwersytetu Poznańskiego 6, 61-614 Poznań, Poland; 4 Sustainable Agricultural Systems Laboratory, USDA-ARS, Henry A. Wallace Beltsville Agricultural Research Center, Beltsville, MD 20705-2350 USA

**Keywords:** Environmental pollutants, Filamentous eukaryotic pathogens, Fungal bioremediation, Heavy metal toxicity and detoxification, Hormesis, Pathogenicity

## Abstract

Heavy metal (HM) contamination of the environment is a major problem worldwide. The rate of global deposition of HMs in soil has dramatically increased over the past two centuries and there of facilitated their rapid accumulation also in living systems. Although the effects of HMs on plants, animals and humans have been extensively studied, yet little is known about their effects on the (patho)biology of the microorganisms belonging to a unique group of filamentous eukaryotic pathogens, i.e., fungi and oomycetes. Much of the literature concerning mainly model species has revealed that HM stress affects their hyphal growth, morphology, and sporulation. Toxicity at cellular level leads to disturbance of redox homeostasis manifested by the formation of nitro-oxidative intermediates and to the induction of antioxidant machinery. Despite such adverse effects, published data is indicative of the fact that fungal and oomycete pathogens have a relatively high tolerance to HMs in comparison to other groups of microbes such as bacteria. Likely, these pathogens may harbor a network of detoxification mechanisms that ensure their survival in a highly HM-polluted (micro)habitat. Such a network may include extracellular HMs immobilization, biosorption to cell wall, and/or their intracellular sequestration to proteins or other ligands. HMs may also induce a hormesis-like phenomenon allowing the pathogens to maintain or even increase fitness against chemical challenges. Different scenarios linking HMs stress and modification of the microorganisms pathogenicity are disscused in this review.

## INTRODUCTION

Fungi and oomycetes represent eukaryotic microbes that are characterized by filamentous vegetative hyphae networks, form spores for asexual and sexual reproduction and share similar processes of infection and nutrition acquisition (Richards et al. 2006). Nonetheless, some features distinguish oomycetes from fungi. Cell walls of oomycetes are composed mainly of β-glucans and hydroxyproline while chitin is the main component of true fungal cell walls. Fungi are haploid or dikaryotic during the major part of their lifecycle, and form septate hyphae. Oomycetes are diploid during their vegetative stage, form coenocytic (nonseptate) hyphae, and produce motile zoospores with two kinds of flagella (Latijnhouwers et al. 2003; Rossman and Palm 2006). As opposed to fungi, which synthesize lysine de novo via α-aminoadipate pathway, oomycetes synthesize the amino acid by α,ε-diaminopimelic acid pathway. Moreover, many oomycetes are (partial) sterol auxotrophs (Latijnhouwers et al. 2003). Even though oomycetes appear fungus-like, they are classified as stramenopiles along with brown algae and diatoms (Beakes et al. 2012). In spite of the distinct evolutionary origin, both fungal and fungal-like pathogens inhabit comparable ecological niches, and many of them cause plant and animal diseases. Importantly, the jeopardy posed by these filamentous eukaryotic pathogens is enhanced by accelerated pathogen evolution, due mainly to the use of fungicides and other human-dependent activities that contribute to the influx of toxic compounds in the microbial environment, often containing heavy metals (HMs) (Pandaranayaka et al. 2019).

### Heavy metals in the microbial environment

Heavy metals are defined as metallic elements with high density and high toxicity to living (micro)organisms. They include two groups: (1) essential elements that in small amounts are crucial for the physiological functions of organisms but toxic when present in excess [*e.g.*, chromium (Cr), iron (Fe), zinc (Zn)] and (2) non-essential elements of an unknown biological role but adversely affect the organism [e.g., cadmium (Cd), lead (Pb), mercury (Hg)]. HMs occur naturally in the environment, the natural sources include weathering of metal-containing rocks and volcanic eruptions. The increase in agricultural and industrial activities has dramatically accelerated the environmental pollution due to HMs while the anthropogenic origins have contributed to the dispersal of HMs in soil, water and air (Callender 2003). Moreover, HMs can spread over long distances in both gaseous and solid phase forms, facilitating their rapid accumulation not only in soil and water but also in some living systems. For example, soils with high HMs concentrations are absorbed by and accumulate in plants, which are eventually transferred to animals and humans via food chain (Zhuang et al. 2013, 2014). Plants, animals and humans as potential hosts can lead to a HM-polluted microenvironments where pathogens dynamically adjust to survive. Thus, soil and/or water pollution by HMs is a critical and complex-dynamic environmental problem.

Even though HMs are among the most investigated environmental pollutants, little is known about their effects on (patho)biology of fungi and fungal-like organisms. Knowledge about the latter is crucial and important since the filamentous eukaryotic pathogens include causal agents of many destructive diseases and the risk of the HMs to target pathogenicity-related events. In this review, we discuss various aspects of HMs toxicity and links between HMs stress and pathogenicity of microorganisms.

### Toxic effects of HMs on filamentous pathogens

Heavy metals such as copper (Cu), manganese (Mn), molybdenum (Mo), Fe and Zn are recognized as being essential for growth and development of plants (Arif et al. 2016; Singh et al. 2016) as well as for the maintenance of various biochemical and physiological functions in humans and animals (Hejna et al. 2019); however, all metals in excessive concentrations are harmful to living organisms. For instance, HMs can complex with molecules such as proteins and lead to their inactivation (Gadd 1993; Singh et al. 2015). In relation to the non-pathogenic microorganisms inhabiting the soil, the research has revealed that essential HMs may provoke significant growth inhibition as well as morphological and physiological changes (Roane 1999; Maanan et al. 2015; Wu et al. 2016). Moreover, metals at elevated concentrations have a negative effect on the soil microbial population and their activities associated with soil respiration rate, which may also contribute to a reduction in soil fertility (Smith 1996). In contrast to essential HMs, effects of non-essential ones on both non-pathogenic and pathogenic microorganisms are poorly recognized and mostly unknown. However, it is becoming an increasingly important research focus in light of the increasing environmental pollution.

In general, soil organisms exhibit high tolerance to HMs acquired likely through evolutionary adaptation to contaminated environment. Fungi are considered to be more tolerant to environmental HMs than other microorganisms, for instance bacteria, because of differences in the cellular metabolism (Rajapaksha et al. 2004; Mocek-Płóciniak 2011). Higher osmotic pressure in the cell structure of fungi allows them to survive adverse conditions (Mocek-Płóciniak 2011). Moreover, fungi can survive in the soil as sclerotia, chlamydospores, or other structures that allow the microorganisms to survive under unfavorable conditions (Golubović-Ćurguz 2010). High tolerance of fungi has been observed when the tolerance threshold to Cu and Zn of pure cultures of systematically distant soil microorganisms were compared. At high Zn and Cu concentrations (128 mmol kg ^−1^) separately applied to growing media, fungal activity (acetate-in-ergosterol incorporation rate) increased by 3 and 7 times, respectively, as compared to the control. At higher levels of HM contamination, a gradual ~ 90% decrease in bacterial activity measured as thymidine incorporation rate was observed (Rajapaksha et al. 2004).

In vitro, human pathogenic yeasts such as *Cryptococcus neoformans* and *Candida albicans* were found resistant to Cu ions. *C. neoformans* H99 was found resistant to ~ 2 mM Cu in liquid medium while clinical isolates of *C. albicans* were able to tolerate ~ 20 mM Cu (Ding et al. 2013). The interactions between fungi or fungal-like microorganisms and HMs are dependant upon several factors, the most significant being their degree of tolerance and absorbtion of HMs ions from soil (Golubović-Ćurguz 2010). Some fungal species display a high tolerance threshold to environments contaminated or polluted by a particular HM or a specific group of HMs (Anahid et al. 2011; Oladipo et al. 2018). For example, *Aspergillus foetidus* had lowest degree of tolerance to HMs such as nickel (Ni), cobalt (Co) or Zn while, similar to *Aspergillus niger*, it revealed a relatively high tolerance to Mo and vanadium (V) (Anahid et al. 2011). Moreover, *Rhizopus microsporus* was found tolerant to a wide range of Cu, Pb and Fe concentrations (400–1000 mg kg^−1^); however, its high tolerance capacity was apparent only at 25 mg kg^−1^ of Cd and 125 mg kg^−1^ of arsenic (As) (Oladipo et al. 2018).

The toxicity of HMs toward fungi and fungal-like microorganisms manifests mainly at morphological level (Baldrian 2003; Vashistha and Chaunhary 2019). In turn, the HM-dependent response of filamentous pathogens at cellular level is similar to that in animals and plants since these toxic elements provoke oxidative damage and induce antioxidant machinery (Table 1).

**Table 1:** The toxic effect of HMs on filamentous pathogens

Metal	Pathogen group	Concentration	Species	Disease(s)	Effect(s)	References
Ag	Fungi	15 mg·L^−1^	*Alternaria alternata*	Leaf spot, black spot	- Growth inhibition - Damage and deformation of hyphae and conidia - Disorders in the total content of protein/lipids/sugar/n-acetyl glucosamine	Ouda (2014)
			*Botrytis cinerea*	Gray mold		
		5–250 ppm	*Sclerotinia sclerotiorum*	White mold	- Growth inhibition	Mwangi et al. (2014)
	Oomycetes	5–50 ppm	*Phytophthora* spp.	Soil- and water-borne plant pathogens	- Zoospore death	Slade and Pegg (1993)
Ba	Fungi	10^–4^ to 10^–5^ M	*Alternaria solani*	Early blight	- Inhibition of spore germination - Inhibition of germ tube growth	Bhajbhuje (2013)
Cd	Fungi	10^–4^ to 10^–5^ M	*Alternaria solani*	Early blight	- Inhibition of spore germination - Inhibition of germ tube growth	Bhajbhuje (2013)
		200 mg·L^−1^	*Aspergillus fumigatus*	Aspergillosis	- Hyphae decolorisation	Mohammadian Fazli et al. (2015)
		0.175–3.0 mM	*Botrytis cinerea*	Gray mold	- Growth inhibition - Over-accumulation of hydrolases and oxidases	Cherrad et al. (2012)
		100 ppm	*Fusarium oxysporum*	Fusarium wilt	- Growth inhibition	Golubović-Ćurguz (2010)
		0.150 mM	*Fusarium oxysporum*	Fusarium wilt	- Acquisition orange tones of mycelium	Lorenzo-Gutiérrez et al. (2019)
		0.1 mM	*Heliscus lugdunensis (Neonectria lugdunensis)*	Black foot	- Growth inhibition (50% decrease)	Jaeckel et al. (2005)
		200 mg·L^−1^	*Paecilomyces* sp.	Oculomycosis Pistachio dieback Entomopathogenic species	- Hyphae decolorisation	Mohammadian Fazli et al. (2015)
		50 μM	*Phanerochaete chrysosporium*	White-rot (woody plants) Granulomatous lung disease	- Oxidative stress induction -Time-dependent up-regulation of CAT, POX, LiP, and MnP activities	Zhang et al. (2015)
		1–500 μM	*Phanerochaete chrysosporium*	White-rot (woody plants) Granulomatous lung disease	- Viability reduction - CAT activity up-regulation	Chen et al. (2014)
		0.1 mM	*Rhizopus arrhizus*	Rhizopus soft rot mucormycosis	- Reduction in hyphae length - Reduction in the number of branches	Gadd et al. (2001)
		0.1–0.2 mM	*Schizophyllum commune*	Sap rot, Schizophyllum rot fungal sinusitis	- Inhibition of radial growth	Lilly et al. (1992)
		5–250 ppm	*Sclerotinia sclerotiorum*	White mold	- Growth inhibition	Mwangi et al. (2014)
		1, 5 and 10 mmol/L	*Trichosporon cutaneum*	White piedra	- SOD activity up-regulation	Lazarova et al. (2014)
		0.7 mM	*Verticillium cf. alboatrum*	Verticillium wilt	- Growth inhibition (30% decrease)	Jaeckel et al. (2005)
	Oomycetes	0.05–3.0 mM	*Achlya bisexualis*	Saprolegniasis in fishes	- Decrease in mycelial area and radial extension	Lundy et al. (2001)
		5 and 12.5 mg/L	*Phytophthora infestans*	Late blight	- ROS and RNS formation - Increase in protein carbonylation content - Up-regulation of CAT and SOD activities - Nitro-oxidative modifications of proteins and nucleic acids	Gajewska et al. (2020)
		10–50 µg/ml	*Saprolegnia delica* Coker	Saprolegniasis in fishes	- Inhibition of sporangia formation - Morphological abnormality (*e.g.* thicker and stunted vegetative hyphae, shorter and thicker zoosporangia, low numbers of oogonia and antheridia)	Ali (2007)
Cu	Fungi	25–100 ppm	*Alternaria alternata*	Leaf spot, black spot	- Increase in total protein content - CAT activity up-regulation	Shoaib et al. (2015)
		15 mg·L^−1^	*Alternaria alternata*	Leaf spot, black spot	- Growth inhibition - Damage and deformation of hyphae and conidia - Disorders in the total content of protein, lipids, sugar and n-acetyl glucosamine	Ouda (2014)
		10^–4^ to 10^–5^ M	*Alternaria solani*	Early blight	- Inhibition of spore germination - Inhibition of germ tube growth	Bhajbhuje (2013)
		0.5 mM	*Aspergillus niger*	Black mold	- Growth inhibition - Nitrate-dependent induction of oxalic acid production	Sazanova et al. (2015)
		20 and 40 ppm	*Aspergilus niger*	Black mold Pneumonia	- Growth inhibition - Decrease in the colony numbers	Abu-Mejdad (2013)
		15 mg·L^−1^	*Botrytis cinerea*	Gray mold	- Growth inhibition - Damage and deformation of hyphae and conidia - Disorders in the total content of protein, lipids, sugar and n-acetyl glucosamine	Ouda (2014)
		0.5 mM	*Penicillium citrinum*	Yellow rice disease (citrinin production) a tissue-invasive cause of pneumonia	- Growth inhibition - Nitrate-dependent induction of oxalic acid production	Sazanova et al. (2015)
		0.1 mM	*Rhizopus arrhizus*	Rhizopus soft rot mucormycosis	- Reduction of hyphae length - Reduction in the number of branches	Gadd et al. (2001)
		1, 5, 3 mmol/L	*Trichosporon cutaneum*	White piedra	- Oxidative stress induction - SOD activity up-regulation	Lazarova et al. (2014)
	Oomycetes	0.05–3.0 mM	*Achlya bisexualis*	Saprolegniasis in fishes	- Decrease in mycelial area and radial extension	Lundy et al. (2001)
		0.5–1 mM	*Phytophthora capsici*	Blight and fruit rot of peppers	- Growth inhibition - Limited sporulation	Liu et al. (2018)
Co	Oomycetes	0.05–3.0 mM	*Achlya bisexualis*	Saprolegniasis in fishes	- Decrease in mycelial area and radial extension	Lundy et al. (2001)
Cr	Fungi	10^–4^ to 10^–5^ M	*Alternaria solani*	Early blight	- Inhibition of spore germination - Inhibition of germ tube growth	Bhajbhuje (2013)
		1, 5 and 10 mmol/L	*Trichosporon cutaneum*	White piedra	- Oxidative stress induction - SOD activity up-regulation	Lazarova et al. (2014)
	Oomycetes	0.5–1 mM	*Phytophthora capsici*	Blight and fruit rot of peppers	- Growth inhibition - Limited sporulation	Liu et al. (2018)
Fe	Fungi	10^–4^ to 10^–5^ M	*Alternaria solani*	Early blight	- Inhibition of spore germination - Inhibition of germ tube growth	Bhajbhuje (2013)
Hg	Fungi	10^–4^ to 10^–5^ M	*Alternaria solani*	Early blight	- Inhibition of spore germination - Inhibition of germ tube growth	Bhajbhuje (2013)
		5–250 ppm	*Sclerotinia sclerotiorum*	White mold	- Growth inhibition	Mwangi et al. (2014)
	Oomycetes	3 mM	*Achlya bisexualis*	Saprolegniasis in fishes	- Decrease in mycelial area and radial extension - Spiral growth of hyphae	Lundy et al. (2001)
		0.5–1 mM	*Phytophthora capsici*	Blight and fruit rot of peppers	- Growth inhibition - Limited sporulation	Liu et al. (2018)
		1 ppm	*Phytophthora nicotianae* var. parasitica	Black shank	- Reduction in zoospore germination	Slade and Pegg (1993)
Li	Oomycetes	1 ppm	*Phytophthora nicotianae* var. parasitica	Black shank	- Reduction in zoospore germination	Slade and Pegg (1993)
Mn	Oomycetes	0.1–200 mg/L	*Phytophthora nicotianae*	Black shank	- Growth inhibition - Sporangiogenesis and zoosporogenesis inhibition - Spores germination inhibition - Concentration-dependent regulation of SOD and CAT activity - Increased MDA content with increasing HM concentration	Luo et al. (2020)
Pb	Fungi	25 μM	*Phanerochaete chrysosporium*	White-rot (woody plants) Granulomatous lung disease	- ROS formation - Time-dependent up-regulation of CAT, POD, LiP and MnP activities	Zhang et al. (2015)
	Oomycetes	1 ppm	*Phytophthora nicotianae* var. parasitica	Black shank	- Reduction in zoospore germination	Slade and Pegg (1993)
		10–50 µg/ml	*Saprolegnia delica* Coker	Saprolegniasis in fishes	- Inhibition of sporangia formation - Morphological abnormality (*e.g.* thicker and stunted vegetative hyphae, shorter and thicker zoosporangia, low numbers of oogonia and antheridia)	Ali (2007)
Sr	Oomycetes	30 mM	*Phytophthora cinnamomi*	Root rot and cankering	- Encystment 90% of zoospores	Byrt et al. (1982)
Zn	Fungi	20 and 40 ppm	*Aspergilus niger*	Black mold	- Growth inhibition - Decrease in the colony numbers	Abu-Mejdad (2013)
		2 mM	*Aspergillus niger*	Black mold	- Growth inhibition - Nitrate-dependent induction of oxalic acid production	Sazanova et al. (2015)
		1–10 mM	*Penicillium* spp.	Allergic pulmonary disease	- Secretion a yellow substance related to the HM stress	Ezzouhri et al. (2009)
		2 mM	*Penicillium citrinum*	Yellow rice disease (citrinin production) a tissue-invasive cause of Pneumonia	- Growth inhibition - Nitrate-dependent induction of oxalic acid production	Sazanova et al. (2015)
		0.1 mM	*Rhizopus arrhizus*	Mucormycosis	- Reduction of hyphae length - Reduction in the number of branches	Gadd et al. (2001)
		5–250 ppm	*Sclerotinia sclerotiorum*	White mold	- Growth inhibition	Mwangi et al. (2014)
	Oomycetes	0.05–3.0 mM	*Achlya bisexualis*	Saprolegniasis in fishes	- Decrease in mycelial area and radial extension	Lundy et al. (2001)
		0.1–20 mg/L	*Phytophthora nicotianae*	Black shank	- Growth inhibition - Sporangiogenesis and zoosporogenesis inhibition - Spores germination inhibition - Concentration-dependent regulation of SOD and CAT activity - Increased MDA content with Increasing HM concentration	Luo et al. (2020)

### Pathogen viability and morphological disorders

HM-mediated changes in mycelial growth and morphological disorders of mycelium are the most visible effects of their toxicity. HMs can also provoke changes in viability and sporulation of phytopathogenic microorganisms such as *Phanerochaete chrysosporium* (Chen et al. 2014), *Botrytis cinerea*, *Alternaria alternata* (Ouda 2014), *Phytophthora capsici* (Liu et al. 2018) and *Phytophthora infestans* (Gajewska et al. 2020).

Undoubtedly, Cd is the most investigated non-essential HM that affects radial growth of the basidiomycete and ascomycete fungi including *Schizophyllum commune* (Lilly et al. 1992) and *Fusarium oxysporum* Schlecht., respectively. The strongest inhibitory effect was found at 100 ppm Cd (Golubović-Ćurguz 2010). However, 0.1 mM Cd diminished not only hyphae length but also the number of branches in *Rhizopus arrhizus*. Also, in *Trichoderma viride*, a fungus which is commonly used as a biofungicide, Cd and Cu were found to cause disruption in the distribution of the fungal biomass within the colony. Most biomass in the presence of Cd was located at the colony interior while in the presence of Cu it was at the periphery of the colony suggesting different modes of HM translocation (Gadd et al. 2001).

Significant changes in the growth rate in response to Cd exposure were also observed in aquatic hyphomycete *Heliscus lugdunensis* and the terrestrial fungus *Verticillium cf. alboatrum*. Application of 0.1 mM Cd inhibited pathogen growth by 50% in *H*. *lugdunensis* while 0.7 mM Cd inhibited 30% growth of *V. alboatrum* (Jaeckel et al. 2005). Cd treatment was found to markedly diminish the viability of the white rot fungus *P. chrysosporium* (Chen et al. 2014). The reduction of cell viability was observed even at the lowest (1 µM) studied HM concentration, however, the highest percentage of cell death (approx. 74%) occurred at concentrations of 100 and 500 µM of Cd. Exposure of *B. cinerea* to Cd stress in the range of 0.175–3.0 mM led to a strong growth inhibition that correlated with the overaccumulation of hydrolases and oxidases (Cherrad et al. 2012).

Independent treatment with Zn and Cu HMs at 20 and 40 ppm were found to inhibit mycelial growth and colony number of two most common human pathogenic fungi, viz., *Aspergilus niger* and *C. albicans.* In turn, Cu-dependent toxic effect in another human pathogenic yeast *C. neoformans* was observed only at 20 ppm HM (Abu-Mejdad 2013). It has been premised that this effect may be related to the presence of Cu-sensitive metallothionein gene which is induced at excessive Cu ions (Carri et al. 1991). Inhibitory effects by individual treatment with Zn (2 mM) and Cu (0.5 mM) on the growth of *A. niger* and *Penicillium citrinum* have also been observed; however, HM-mediated growth inhibition was significantly alleviated in media supplied with nitrate sources (Sazanova et al. 2015). This effect could be associated with nitrate–mediated oxalic acid production, a compound that detoxifies HMs in fungi (Sazanova et al. 2015). Inhibitory effect of Cd, Hg and silver (Ag) separately appilied to the growth medium of phytopathogen *Sclerotinia sclerotiorum* provoked also opaque halo effect surrounding the culture (Mwangi et al. 2014). In *Alternaria solani* cultures separately treated with variuos HMs [Hg, Cu, barium (Ba), Fe, Cd, lithium (Li)] at concentrations that ranged from 10^–4^ to 10^–5^ M observed the inhibition of germ tube growth and spore germination. The most severe inhibitory effects were found due to Hg, Cd, or Cu (Bhajbhuje 2013). Ag and Cu nanoparticles (15 mg·L^−1^ Ag and Cu) independently tested against plant pathogenic fungi *B. cinerea* and *A. alternata* led to inhibition of their hyphal growth (Ouda 2014). Microscopic observation revealed that growth reduction was accompanied by damage of hyphae and conidia in both the fungi. Biochemical analysis of the culture filtrate revealed that Ag caused reduction in the total content of protein, lipids, sugar, and n-acetyl glucosamine (Ouda 2014).

In addition to the observed HM-mediated morphological changes, the toxic elements led to the formation of colorful or decolorized mycelia, particularly under in vitro conditions. Mycelia of *F. oxysporum* grown under Cd stress acquired orange tones (Lorenzo-Gutiérrez et al. 2019) while Cd-mediated formation of orange-brown pigments was observed in fungal colonies of white rot fungus *Abortiporus biennis* (Jarosz-Wilkołazka et al. 2006). The induction of pigments may be related to Cd biosorption onto the cell walls (Lorenzo-Gutiérrez et al. 2019). On the contrary, decolorization of fungal hyphae at higher Cd concentration in the growth medium was observed in *Paecilomyces* genera (Mohammadian Fazli et al. 2015). *Penicillium* isolates grown on agar media enriched with high Zn concentrations were found to secrete a yellow substance likely related to the HM stress (Ezzouhri et al. 2009).

In fungal-like pathogens such as *Achlya bisexualis*, HM stress caused by individual treatment with Cu, Co, Hg, Zn, or Cd in the range of 0.05–3.0 mM decreased the hyphal area and limited the radial extension of the oomycete. Hg (3 mM) caused abnormal spiral growth of the hyphae (Lundy et al. 2001). A decrease in hyphal growth together with reduced sporulation of *P. capsici,* a cause of blight and fruit rot of peppers, was evident in response to Cu, Cr, or Hg independently applied to the culture (Liu et al. 2018). Cadmium at 5 and 12.5 mg/L also limited the in vitro growth of *P. infestans,* fungus responsible for the late blight disease. The reduced hyphal growth was accompanied by an inhibition of sporangia formation and spore germination (Gajewska et al. 2020). A similar inhibitory effect together with variable morphological abnormalities were observed also in two zoosporic fungi *Saprolegnia delica* Coker and *Dictyuchus carpophorus* Zopf. separately treated with Cd or Pb in range of 10–50 µg/ml (Ali 2007). In another pathogenic oomycete, *Phytophthora cinnamomi,* 30 mM strontium (Sr) caused encystment of 90% zoospores (Byrt et al. 1982). Notably, low concentrations of Hg and Pb (1.0 ppm) applied separately reduced zoospore germination of *Phytophthora nicotianae* var. *parasitica* by over 50% while Ag in the range of 5–50 ppm caused death of most *Phytophthora* spp. zoospores (Slade and Pegg 1993). It is important to note here that response to various metals during the lifecycle of oomycetes *Phytophthora* and *Pythium* revealed that generally metals are toxic at lower concentrations for zoospores than at any other stage of their life cycle (Slade and Pegg 1993).

### Disturbance of the cell redox homeostasis

One of the earliest cellular response to HMs is the generation of reactive oxygen and nitrogen species (ROS/RNS) that result in oxidative and/or nitrosative stress and disturbance of the cell redox balance (Rodríguez-Serrano et al. 2009; Oves et al. 2016; Georgiadou et al. 2018). Cells activate mechanisms that detoxify reactive species and protect cells from oxidative damage. Thus, antioxidant system involving peroxidases (POXs), superoxide dismutase (SOD), and catalase (CAT) get activated (Krishnamurthy and Wadhwani 2012; Ighodaro and Akinloye 2018). However, little is known about any such antioxidant response in pathogenic microorganisms (Pamplona and Constantini, 2011; Caverzan et al. 2016; Kusvuran et al. 2016).

Oxidative stress as a consequence of HM exposure affects white-rot fungus *P. chrysosporium* (Chen et al. 2014; Zhang et al. 2015), *A. alternata* (Shoaib et al. 2015), filamentous yeast *Trichosporon cutaneum* (Lazarova et al. 2014)*,* and oomycete *P. infestans* (Gajewska et al. 2020). In *P. chrysosporium* Pb (25 µM) and Cd (50 µM) independently applied caused ROS formation and time-dependent changes in the activity of CAT, POX, lignin peroxidase (LiP), and manganese peroxidase (MnP). The intracellular enzymes such as CAT and POX showed a similar trend in the activity, however, HM-mediated induction of CAT and POX was lower than that in LiP and MnP (Zhang et al. 2015). Response of *A. alternata* to Cu sulphate salt provoked a visible growth inhibition that correlated with significant increase in the total protein pool and CAT activity (Shoaib et al. 2015). The antioxidant response of pathogenic fungi under HMs stress involves also SOD, which has been shown to be required for full virulence in the models *C. albicans*, *C. neoformans* and *Aspergillus fumigatus* (Warris and Ballou 2019). In general, SOD activity increased in response to individual treatment with Cd, Cr, or Cu in filamentous yeast *T. cutaneum;* however, only Cd treatment resulted in dose-dependent increase in the enzyme activity, the highest induction being noted at 10 mmol/L (Lazarova et al. 2014). The independent treatment of *P. nicotianae* with Mn (0.1–200 mg/L) and Zn (0.1–20 mg/L) provoked elevation of CAT and SOD activities at the selected HM dose (1 mg/L), while concentrations above this threshold gradually diminished the enzyme activity (Luo et al. 2020).

Exposure of *P. infestans* to Cd led to HM-dependent ROS/RNS overproduction and nitro-oxidative modifications of RNA, DNA, and protein pools (Gajewska et al. 2020). Cadmium based dose-dependent RNA’s nitrative modification observed in the oomycete suggested that HM toxicity can contribute to the RNA lesion. However, it is noted here that nitrative and/or oxidative modifications can also reprogram post-transcriptional gene expression via modulation of mRNA levels and changes in the activity of transcription factors (Chmielowska-Bąk et al. 2018). In addition, moderate Cd stress (5 mg/L) can induce antioxidant response manifestation by increasing CAT and SOD activities as well as result in a novel Cd-dependent CAT isoform in *P. infestans* hyphae (Gajewska et al. 2020). Thus, an early activation of antioxidant enzymes might be the first line of defence against Cd toxicity in microorganisms before the induction of specific HM defence (Basha and Rani 2003).

The divergent effects of various HMs on the growth, development, and physiology of pathogenic microorganisms emphasizes that the HM toxicity depends not only on the type of metal, their applied form and concentration but also on fungal/fungal-like life style, species or even isolates.

### The hormetic effect of HMs on pathogens

A low dose of harmful factors can cause a hormesis effect. This phenomenon refers to adaptive responses of organisms to moderate environmental challenges, improving their functionality and/or tolerating stronger challenges in the future (e.g., Kendig et al. 2010; Calabrese and Mattson 2017). Even though the improved performance of some plant and animal species in response to HMs has been observed (Poschenrieder et al. 2013; Jalal et al. 2021), very limited information in microorganisms, particularly fungi and oomycetes, is available about the stimulating effect of HMs on their growth and development (Table 2).

**Table 2:** The hormetic effect of HMs on filamentous pathogens

Metal	Pathogen group	Concentration	Species	Disease(s)	Effect(s)	References
Cd	Oomycetes	3 ppm	*Pythium debaryanum*	Damping-off and seedling disease	- Stimulation of mycelia growth	Golubović-Ćurguz et al. (2010)
Co	Fungi	5 ppm	*Sclerotinia sclerotiorum*	White mold	- Stimulation of mycelia growth	Mwangi et al. (2014)
Cu	Fungi	5 mg L^−1^	*Aspergillus flavus*	Aspergillus ear and Kernel rot human and animal Aspergillosis	- Stimulation of mycelia growth - Increase in total RNA content - Induction of aflatoxin biosynthesis	Cuero et al. (2003)
		5 × 10^–4^ and 5 × 10^–3^ M	*Endothia parasitica*	Chestnut blight	- Stimulation of mycelia growth	Englander and Corden (1971)
		3 ppm	*Fusarium oxysporum*	Fusarium wilt	- Stimulation of mycelia growth	Golubović-Ćurguz et al. (2010)
		5 ppm	*Sclerotinia sclerotiorum*	White mold	- Stimulation of mycelia growth	Mwangi et al. (2014)
	Oomycetes	3 ppm	*Pythium debaryanum*	Damping-off and seedling disease	- Stimulation of mycelia growth	Golubović-Ćurguz et al. (2010)
Fe	Fungi	5 mg L^−1^	*Aspergillus flavus*	Aspergillus ear and Kernel rot human and animal Aspergillosis	- Stimulation of mycelia growth - Increase in total RNA content - Induction of aflatoxin biosynthesis	Cuero et al. (2003)
		5 × 10^–4^ and 5 × 10^–3^ M	*Endothia parasitica*	Chestnut blight	- Stimulation of mycelia growth	Englander and Corden (1971)
Mg	Fungi	20 and 40 ppm	*Aspergillus niger*	Black mold	- Increase in the colony numbers	Abu-Mejdad (2013)
Pb	Oomycetes	3 ppm	*Pythium debaryanum*	Damping-off and seedling disease	- Stimulation of mycelia growth	Golubović-Ćurguz et al. (2010)
Zn	Fungi	5 mg·L^−1^	*Aspergillus flavus*	Aspergillus ear and Kernel rot human and animal Aspergillosis	- Stimulation of mycelia growth - Increase in total RNA content - Induction of aflatoxin biosynthesis	Cuero et al. (2003)
		5 × 10^–4^ and 5 × 10^–3^ M	*Endothia parasitica*	Chestnut blight	- Stimulation of mycelia growth	Englander and Corden (1971)
	Oomycetes	10–30 µg/ml	*Saprolegnia delica* Coker	Saprolegniasis in fishes	- Stimulation of mycelia growth - Sporangia elongation	Ali (2007)

Heavy metals (Cu, Zn, and Fe independently supplied at concentrations of 5 × 10^–4^ and 5 × 10^–3^) stimulate mycelial growth of phytopathogenic fungus *Endothia parasitica* (Englander and Corden 1971). The same metals applied to *A. flavus* at relatively low concentration (5 mg L^−1^) were found to stimulate not only the mycelial growth of the pathogen but also aflatoxin biosynthesis*.* Moreover, the accumulation of total RNA was enhanced by Cu, Zn and Fe; however, the combination of all three HMs or their duplexes (Zn + Cu and Zn + Fe) were much more effective in total RNA accumulation as compared to the control (Cuero et al. 2003). Cu at 40 ppm led to increased growth of *C. neoformans* and exerted a similar effect as magnesium (Mg), although the latter is not an HM (Abu-Mejdad 2013). Among 12 metals independently tested, only Cu and Co stimulated mycelial growth of *S. sclerotiorum* (Mwangi et al. 2014). Also, it has been shown that at low Cu concentration (3 ppm) the growth of phytopathogens such as *Pythium debaryanum* and *F. oxysporum* is stimulated (Golubović-Ćurguz et al. 2010). Also, *P. debaryanum* growth was stimulated in the presence of applied Pb or Cd at a concentration of 3 ppm. The hormetic-like effect of HMs was also shown in two zoosporic fungi, *S. delica* and *D. carpophorus,* in the presence of 10–50 µg/ml Zn (Ali 2007). It was noted that a high number of sporangia were observed in *D. carpophorus* at high Zn concentration (30 µg/ml). Sporangia in both *S. delica* and *D. carpophorus* formed in the presence of Zn were elongated compared to the control (Ali 2007).

### Mechanisms of resistance to heavy metals

**Fig. 1:**
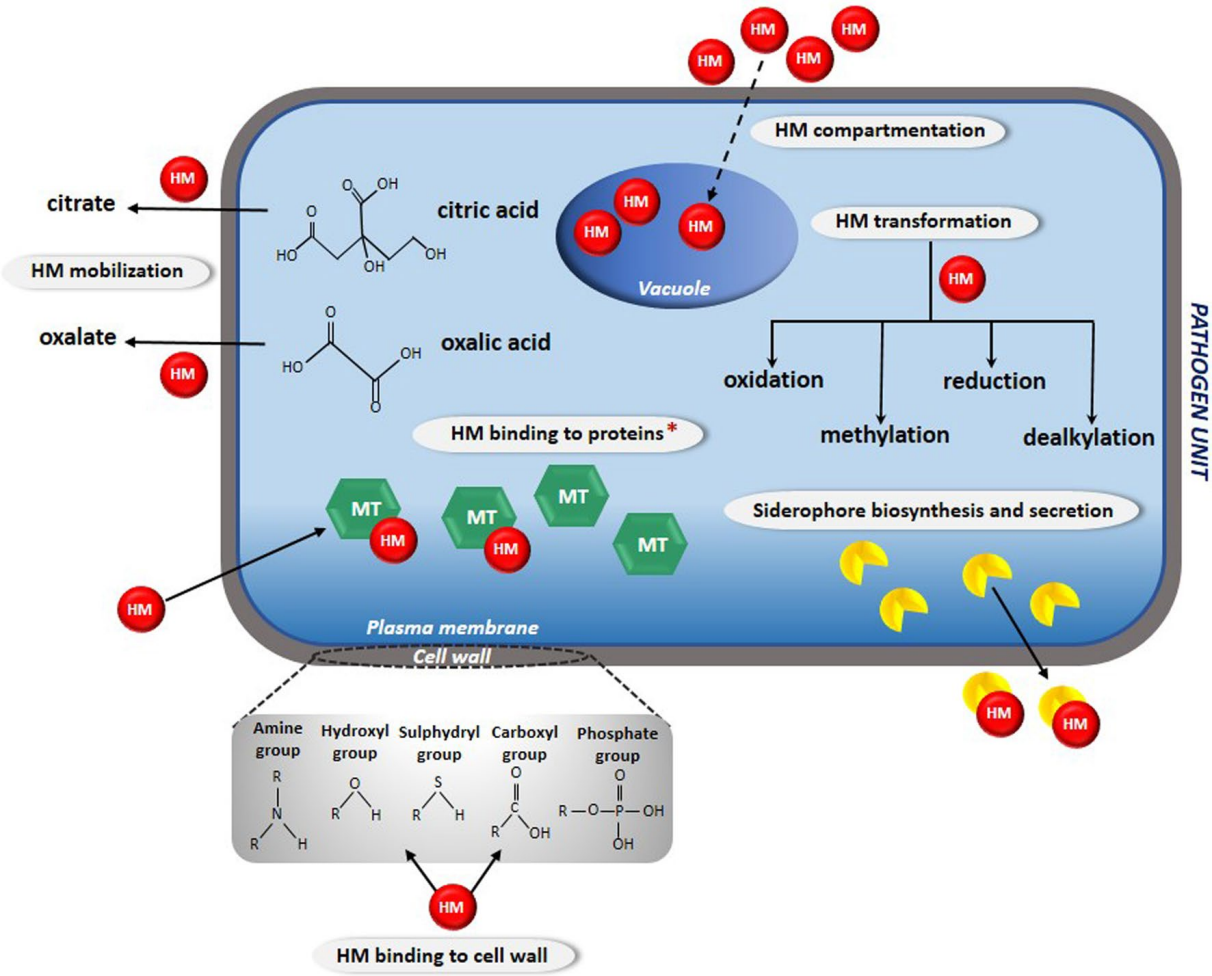
The potential mechanisms of HMs detoxification operating in fungal and fungal-like pathogens. HM heavy metal; MT metallothioneins; Red asterisk indicates the mechanism documented also in oomycetes

Many filamentous eukaryotic pathogens belonging to soil microorganisms exhibit relatively high tolerance threshold to HMs. Most likely, these microorganisms have been subjected to evolution in response to contaminated environments and have developed novel detoxification strategies that allow them to acquire tolerance to pollutants such as HMs (Lorenzo-Gutiérrez et al. 2019). The potential mechanisms that may be involved in the HMs detoxification have been divided into two types (Siddiquee et al. 2015). The first one is based on secretion of chemical compounds outside the cell to bind the metals in the extracellular space or on the cell wall making them biologically inaccessible and therefore less harmful for the cell. The second mechanism occurs when the harmful substances enter the cell; involves the chelation of toxic metal ions in the cytosol, resulting in inactivation and storage of HMs away from sensitive metabolic processes (Avery et al. 1992). Thus, cellular defence of fungi and oomycetes against the excessive concentration of toxic metals in the environment includes intracellular metal sequestration, metal binding to cell walls, chemical transformations and intracellular metal immobilization (Fig. 1) (Gadd 1993; Siddiquee et al. 2015).

### Extracellular metal sequestration

Exposure of microorganisms to HMs may induce synthesis and secretion of chelating molecules that bind to the metal. Fungal metabolites such as organic acids and siderophores may also precipitate metals from the extracellular environment and lead to their inactivation. The model yeast *S. cerevisiae* as an extracellular metal chelator may use hydrogen sulfide to form insoluble metal sulfides (Minney and Quirk 1985).

In general, organic acids may operate outside or within the cell and reduce HM availability by forming stable complexes and insoluble salts (Sazanova et al. 2015). The pH, buffering capacity of the cellular environment, sources of the carbon, phosphorus and nitrogen, all impact the quality, quantity and ability of organic acids to reduce HM (Fomina et al. 2005). For example, *A. niger* and *P. citrinum* grown on a nitrate medium containing Zn enhanced the biosynthesis of oxalic acid, while the addition of Cu stimulated the production of malic acid in both the fungi and citric acid only in *A. niger* (Sazanova et al. 2015). Although under in vitro conditions HMs can stimulate the synthesis of organic acids (*i.e.,* oxalic, citric, succinic, malic, acetic and gluconic), the oxalic acid is more predominant in fungi (Dutton et al. 1993; Clausen et al. 2003; Fomina et al. 2005; Gadd 2010). Supplementation of the growth medium of *F. solani* with Ag (I) at 400 mg/l increased oxalic acid content by 3.5-fold (El Sayed and El-Sayed 2020). The entomopathogenic fungus *Beauveria caledonica* over-excretes acetic, citric and oxalic acids during growth in the presence of singly applied Cd, Cu, Pb or Zn make available crystalline oxalates both extracellularly and within the fungal biomass (Fomina et al. 2005). Oxalate crystals are formed in growth media due to high levels of Zn and Co in white rot fungi *Bjerkandera fumosa*, *Phlebia radiata* and *Trametes versicolor* as well as in the brown rot fungus *Fomitopsis pinicola* (Jarosz-Wilkolazka and Gadd 2003). Phytopathogens can secrete oxalic acid at millimolar concentrations (Lu 2013) that becomes a deterrent to pathogenicity since acidification of host environment facilitates sequestration of calcium ions and consequently the degradation of the host cell walls (Dutton and Evans 1996).

Other organic compounds that chelate HMs extracellularly are siderophores, i.e*.,* low molecular weight ligands that chelate iron and participate in its metabolism in the cell. Siderophores are more efficient at binding metals than most common chelating agents, *for example,* oxalic or citric acids (Holmström et al. 2004). Although the canonical function of siderophores involves iron scavenging, these compounds have also been found to complex with other metals including HMs such as Cd, Cu, Pb, Zn, Ni or As, preventing their uptake by the cell (Ahmed and Holmström 2014). Siderophores produced by *Fusarium solani* were found to contribute to in vitro solubilization of Cu as well as Zn (Hong et al. 2010). The ability to complex metals other than Fe mainly depends on the affinity or selectivity toward individual metals and the stability constants of the resulting metal–siderophore complex (Hernlem et al., 1999). Some phytopathogenic fungi, *i.a., Stemphylium botryosum,* produce unique β-keto aldehydes that function as phytotoxins and also chelate iron (Barash 2014). From chemical point of view fungal siderophores have a hydroxamate type structure with an N-hydroxyornithine moiety. The hydroxamate siderophores are divided into four families including rhodotorulic acid, coprogens, ferrichromes and fusarinines. These compounds are produced by phytopathogens such as *S. botryosum, Epicoccum purpurescens, Ustilago maydis* (Renshaw et al. 2002) as well as zoopathogens belonging to *Fusarium* spp., *Paecilomyces* spp., and *Aspergillus* spp. (reviewed Al-Fakih 2014). In addition to hydroxamate siderophores it is also known that pathogenic fungi may produce polycarboxylates called rhizoferrin from *Rhizopus microsporus* var. *rhizopodiformis* and phenolate-catecholates found in wood-rotting fungi (Larcher et al. 2013). In general, the siderophore production is a result of iron deficiency in the cell (Chennappa et al. 2019). Transport of iron-siderophores is an energy-dependent and stereoselective process depending on the metal ion coordination geometry and the N-acyl residues surrounding the metal centre (Renshaw et al. 2002). Apart from iron transportation and the ability to chelate other toxic metals, siderophores are also engaged in microorganismal pathogenicity including phytopathogens *Cochliobolus miyabeanus*, *C. heterostrophus*, *Fusarium graminearum*, *Alternaria brassicicola* and *Colletotrichum graminicola* (Oide et al. 2006; Albarouki et al. 2014).

### Metal binding to cell walls

The cell wall structure of microorganisms has adapted to bind substances such as HMs and can thereby protect the cells. The cell surface of microorganisms is negatively charged with the presence of various anionic structures, for example, glucan in oomycetes and chitin in fungi (Anahid et al. 2011). Binding of metal to the cell wall can occur through a variety of chemical reactions related to metal biosorption. This type of action prevents the uptake of metal ions into the interior of cells and mainly involves carboxyl and phosphoryl groups; however, biomolecules equipped with amine, hydroxyl, and sulfhydryl groups can also participate in metal binding (Ayangbenro and Babalola 2017). For example, hydroxyl, amide, carboxyl and phosphate-rich cell walls of the lignin-degrading fungus *F. velutipes* exhibit efficient absorption for Cu, Zn and Hg amounting to 73.11%, 66.67% and 69.35%, respectively (Li et al. 2018). Binding of metals to the cell wall is a mechanism that operates at both low and high temperatures, is independent of cellular metabolism, but dependent upon the physicochemical metal parameters such as ionic potential, ionic radius, and ionic stability (Tsekova and Ilieva 2001). In *R. arrhizus*, the most common cause of mucormycosis, the biosorption was independent of the ionic charge but was linearly dependent on ionic radius (Tobin et al. 1984).

### Intracellular metal immobilization and chemical transformations

Metal transporters represent the first line of defense against disturbance in cellular and subcellular homeostasis caused by HMs. In general, ATP-binding cassette (ABC) transporter family is involved in intracellular transfer of HMs in fungi (Kovalchuk and Driessen 2010). Based on the model yeasts, proteins Bpt1p, Hmt1p, Ycf1p, Vmr1p and Nft1p transport HMs and their GSH-dependent conjugates from the cytosol to vacuole. Efflux pumps are considered to be responsible for HMs exclusion. Copper-transporter ATPases, cation diffusion facilitators (CDF), and multidrug and toxin extrusion (MATE) carrier family are among those that exclude non-essential HMs such as Pb, Cd and Ni (Sharma et al. 2021). However, the process requires large amounts of energy causing ATP deficiency that can lead to cell death (Norris and Kelly 1977).

Intracellular immobilization of metals involves their complexation by cytoplasmic compounds that convert potentially toxic metals into less or non-toxic forms and which may then be compartmentalized in the vacuole. Three major classes of intracellular peptide chelating metal ions include glutathione, phytochelatin’s (PCs) and metallothionein’s (MTs). Being thiol compounds they are the prime agents for cellular HMs tolerance and involvement of MTs in fungal HM detoxification. Metallothioneins are low molecular weight cysteine rich proteins that coordinately bind divalent or monovalent metal ions and control the homeostasis of essential metals such as Zn and Cu, or lead to sequestration of the non-essential toxic metals such as Cd (Bellion et al. 2006). MTs are known to have a low degree of sequence similarity. Fungal MTs are distributed into six different families (MTs family numbers F08-F13). However, some MT sequences are not affiliated with known MT family, such as *Basidiomycota* (Ziller and Fraissinet-Tachet 2018). The genera of microbial eukaryotes in which MTs have been predicted to be present include phytopathogenic oomycetes, *i.e., Phytophthora* and *Peronospora* (Balzano et al. 2020). Fungal MT genes can be induced by a single or even multiple metals (Reddy et al. 2014). The detoxification property of MTs against different metals, *e.g.,* Cd and Zn, is seen only in the presence of Cu which triggers MTs production, for example, in cellular environment of *S. cerevisiae* (Calvo et al. 2017).

A single metallothionein (mt1) was found in the genome of *F. oxysporum* that involves resistance to metals and also to fungal pathogenicity (Lorenzo-Gutiérrez et al. 2019). Functional studies have shown that mutants of *mt1* gene were less resistant to the heavy metals such as Cu, Zn, or Cd as compared to the wild-type pathogen. Moreover, *mt1* was found to be transcriptionally regulated specifically by Zn but not by either Cd or Cu. Thus, Mt1 seems to play an important role in Zn homeostasis. In addition, knockout strain for the *mt1* gene revealed that the inability to synthesize Mt1 protein did not affect the virulence of *F. oxysporum* toward tomato and mouse hosts but reduced fungal survival within the phagosome, a membrane-bound vesicle that encloses particulate matter taken into the cell by phagocytosis. The effect was ascribed to impaired resistance of the mutant to ROS and metal ions that could perform antimicrobial functions inside the phagolysosome (Lorenzo-Gutiérrez et al. 2019).

Other types of intracellular metal inactivation involve chemical reactions such as oxidation, reduction, methylation, or dealkylation. One such reaction for fungal microorganisms involves the reduction of Ag (Osorio-Echavarría et al. 2021) and Cu (Antsotegi-Uskola et al. 2020). Fungal endophytes such as *Lindgomycetaceae* P87 and *Curvularia geniculata* P1 were found to reduce mercury ion Hg (II) and the reaction led to the formation of volatile forms of Hg enabling its evaporation (Pietro-Souza et al. 2020). Another example of converting toxic metals to relatively safe compounds is the reduction of Cr (VI) by *A. niger* (Gu et al. 2015)*.* This involves two steps: (i) adsorption of Cr (VI) by carboxyl, hydroxide, amine, amide, cyano and phosphate groups of cell wall, and (ii) the Cr (VI) reduction to Cr (III), which is sparingly soluble in water form, and therefore not toxic to the cell (Gu et al. 2015).

### Effects of HM on pathogenicity

**Fig. 2:**
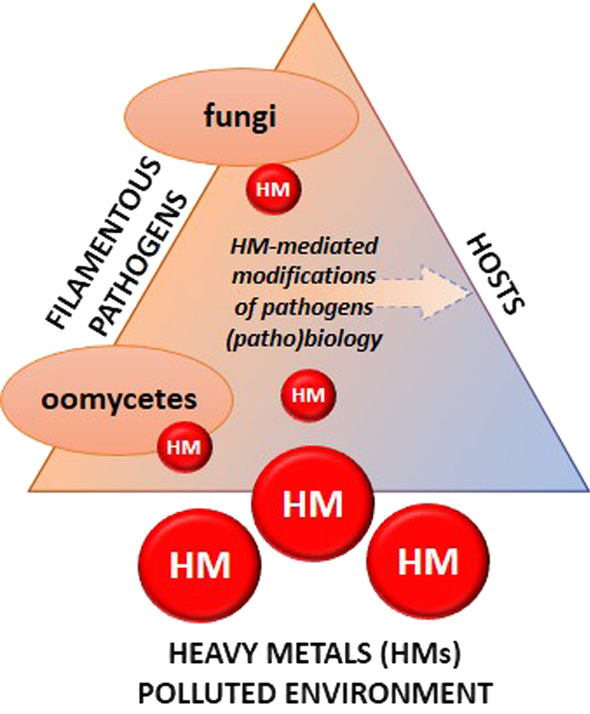
A model linking HMs stress and modification of the microorganisms pathogenicity. Both fungal and fungal-like pathogens inhabit comparable aquatic and soil habitats. Thus, environment pollution, and especially soil or water contamination with HMs can significantly affect the pathogens capability to infect host organisms

Heavy metal polluted environment is well documented risk to human health since contaminants can move relatively freely from soils or water to plants and animals, but the additional risk also arises as a result of tripartite interaction involving HM-pathogen-host system (Fig. 2). It has previously been underlined that some abiotic factors such as high availability of nitrogen or high soil moisture content can increase disease susceptibility by promoting pathogen growth (Samaddar et al. 2021). It is still unclear whether HMs action is related to pathogenicity and/or virulence of the microorganism. Based on the available literature HMs can limit as well as promote pathogen virulence (e.g., Cherrad et al. 2012; Bakti et al. 2018; Liu et al. 2018; Gajewska et al. 2020). These effects indicate that environmental pollution and soil contamination due to HMs can significantly affect the pathobiology of microorganisms, and thus can have huge economic consequences especially in relation to plant pathogens. Thus far, limited reports on the effects of HMs on phytopathogens are available. Usually, altered pathogenicity of fungal and fungal-like pathogens seem connected with enzymes such as proteases involved in infection and effector-related protein secretion (Dong et al. 2012; Williamson 2016). HMs stress related to singly applied Cu, Cr or Hg at low and neutral pH were found to limit the virulence of *P. capsici* (Liu et al. 2018). The observed decrease of *P. capsici* virulence during infection of pepper leaves was associated with reduced expression of two pathogenicity-related genes, *i.e.,* laccase *PcLAC2* and necrosis-inducing NLP protein *PcNLP14.* In *B. cinerea* secretome high levels of two Zn-metalloproteases was noted after the independent pathogen treatment with Cu and Zn. Importantly, these Zn-metalloproteases belong to the group of extracellular peptidases of the deuterolysin and penicillolysin M35 family and are known to be the main virulence factors in *Aeromonas salmonicida* (Cherrad et al. 2012). In the oomycete phytopathogen *P. infestans*, Cd at a concentration of 5 mg/L accentuated pathogenicity, which was manifested by an acceleration of disease symptoms on two out of three potato cultivars, *i.e.*, Bintje, Bzura and Sarpo Mira. Moreover, molecular assessment of disease progression measured as *P. infestans* Tef1 gene (*PiTef1*) expression revealed an elevated *PiTef1* gene expression *in planta* in each tested potato cultivar; however, the level of *PiTef1* expression depended on the degree of basal resistance of the potato cultivars used in this study (Gajewska et al. 2020).

Effects of HMs on pathogenicity regarding human and animal pathogens are better documented. For example, at high concentrations of Cd, Pb, Ni, Cu, or Zn separately added to the media, pathogenicity of *Paecilomyces fumosoroseus* decreased. In contrast, cultures enriched with Mg ions, a non-HM metal, led to increased pathogenicity of this fungus (Jaworska et al. 1998). Another species belonging to *Paecilomyces (P. farinosus)* showed limited pathogenicity in the presence of Cd or Pb ions, whereas independently applied Cu, Zn, or Ni ions each had a weak or no effect on the fungal pathogenicity (Ropek and Para, 2003). Cd also limited pathogenicity of the entomopathogen fungus *Isaria javanica.* Under normal conditions, *I. javanica* caused mortality of two aphid species while after Cd exposure the pathogen caused 30–60% mortality of aphids (Hassn et al. 2014).

It is known that a relationship exists between pathogenicity and oxidative stress tolerance (Bakti et al. 2018; Warris and Ballou 2019). For example, fungi such as *C. albicans*, *C. neoformans* and *Aspergillus fumigatus* that are deficient in SOD exhibit reduced pathogenicity*.* SODs are required for full virulence while defects in oxidative stress response pathways attenuate fungal resistance to phagocyte killing (Warris and Ballou 2019). In *A. fumigatus*, PIB-type cation ATPase (PcaA), a metal transporter with potential to eliminate superoxide radicals, can be involved in metal metabolism and virulence. Also, deficiency of PcaA leads to decreased Cd tolerance and attenuates the virulence of *A. fumigatus* in the *Galleria mellonella* infection model, resulting in a decreased tolerance to oxidative stress (Bakti et al. 2018). The *B*. *cinerea* P-type ATPase BcCcc2, an ortholog of the *S*. *cerevisiae* Ccc2 copper transporting P-type ATPase, was found to be essential for virulence in the necrotroph (Saitoh et al. 2010). Apart from morphogenetic defects caused by the *Bcccc2* deletion, the mutant strain was unable to penetrate and infect tomato leaves and carnation petals. Since the P-type ATPase is engaged in Cu delivery to the secretory compartment for subsequent protein modification, the BcCcc2 targeted proteins were deficient in copper, which resulted in impaired cellular processes (Saitoh et al. 2010). The lack of BcCcc2 can also result in a defective BcSod1 function while preventing the maintenance of the O_2_^−^/H_2_O_2_ ratio during *in planta* development that promotes necrotic lesion formation and disease progression (Antsotegi-Uskola et al. 2020).

Another link between pathogenicity and HMs includes fungal metal detoxification machinery. It is known that the host can mobilize Cu ions as an innate anti-fungal defense to which the pathogen responds by activating specific mechanisms to counteract metal overaccumulation. For instance, human fungal pathogen *C. neoformans* induces genes encoding the Cu-detoxifying metallothionein (Cmt) proteins during pulmonary infection (Ding et al. 2013). *C. neoformans* mutant strains lacking metallothionein genes *CMTs* or expressing Cmt protein variants defective in Cu-coordination have been shown to exhibit severely attenuated virulence and reduced pulmonary colonization (Ding et al. 2013). In relation to plant pathogens, deletion of *Magnaporthe metallothionein 1 (Mmt1)* encoding MT-like protein resulted in diminished pathogenicity of hemibiotroph *Magnaporthe grisea* in rice that had a defect in the pre-penetration phase (Tucker et al. 2004). Mmt1 protein is localized in infection structures such as cell walls of the appressorium and germ tube tips before appressorium development, which suggests its role in cell wall remodeling and hyperosmotic stress adaptation. In addition, Mmt1 possesses the capacity to act as an antioxidant with a very low redox potential allowing the fungus to withstand host oxidative defense (Tucker et al. 2004).

### Potential of pathogenic fungi in HM bioremediation

The question “whether pathogenic microorganisms can be utilized for bioremediation purpose” is quite controversial. Nonetheless, there is good experimental evidence which implies that both non-pathogenic and pathogenic fungi have a good capacity for metal uptake and recovery. In addition, fungi present some features including hyphal growth that confer higher competitiveness in a general bioremediation process such that fungi grow at a faster rate. Also, the production of resilient extracellular enzymes and acids can contribute to a more efficient bioremediation and lead to a more general ability to withstand unfavorable environmental conditions (Blasi et al. 2016). Environmental research has shown that many fungal species, for example*, Trichoderma autroviride*, *T. harzianum*, and *T. virens* as biocontrol agents can remediate sites contaminated with HMs. The fungal remediation principle is based on the endogenous ability of these microorganisms to detoxify metals via biosorption, bioconcentration, and biotransformation. Nonetheless, bioremediation is a complex process influenced mainly by pH and temperature, which can determine the fungal ability to grow, uptake and store metals (Liu et al. 2017). Changes in pH can affect the development of fungal colonies. Moreover, suboptimal pH range may reduce activity of enzymes involved in HM resistance mechanisms, and lead to either low or high bioavailability of the metal (Carrillo-Chávez et al. 2014; Li et al. 2019). Like with pH variations, temperature fluctuations can significantly modify chemistry of toxic elements by reducing or increasing their bioavailability and thereby affect fungal activity and community structure. For example, a temperature of 30 °C was found congenial for HMs removal from a multi metal mixture [containing 6 mg/L of Cu (II), Cr (VI), Cd (II), Zn (II) and Ni (II) each] via entomopathogenic fungus *Beauveria bassiana*. This congenial effect was ascribed to increased biomass production which provided more metal-binding sites (Gola et al. 2016).

Potential of pathogenic fungal species in bioremediation of environment contamination with HMs such as Pb, Cr, Ni and Ag has been experimentally verified in *Fusarium* sp., *Penicillium* sp. and *Aspergillus* sp. (Iram et al. 2013). Also, mycelia of *Rhizopus* and *Absidia* are excellent biosorbents for Pb, Cd, Cu and Zn (Volesky 1994). However, the use of live microorganisms, especially pathogenic ones, in ecological niches may not only be problematic but also extremely controversial. However, it is important to note that mechanisms of HMs biosorption also operate in thermally deactivated microorganisms. Thus, research on *A. flavus* showed that thermal inactivated biomass was also suitable for use as a biosorbent to remove As (III) from aqueous solution (Maheswari and Murugesan 2011). It has also been documented that dead fungal biomass of *A. niger*, *R. oryzae*, and *P. chrysogenum* can be used to convert toxic Cr (VI) to less toxic or nontoxic Cr (III) (Park et al. 2005). Also, the dead biomass of *F. flocciferum* was found to bio-absorb Cu, Cd and Ni (Blessy et al. 2015).

Fungi can remediate environmental contamination due to HMs or metalloids as well as by polycyclic aromatic hydrocarbons (PAHs). Utilization of a heterogeneous group of microorganisms can be particularly helpful in remediating co-contaminated environments that are a serious global problem since HMs are also found in petroleum-contaminated soils. Thus*, F. solani* isolated from petrol station soil was able to degrade more than 60% of the supplied pyrene and accumulate significant amounts of Cu and Zn (Hong et al. 2010). Fungal siderophores in co-contaminated soil could play an important role not only by binding metals other than Fe (III), *e.g*., Cd, Cu, Ni, Pb, Zn, thorium (Th) (IV), uranium (U) (IV), and plutonium (Pu) (IV) but also by facilitating the biodegradation of petroleum hydrocarbons (Ahmed and Holmström 2014; Li et al. 2020). In general, various fungal taxa including *Amorphoteca, Neosartorya, Talaromyces, Aspergillus, Fusarium, Paecilomyces, Sporobolomyces, Cephalosporium, Penicillium,* and *Graphium* have the potential to degrade petroleum hydrocarbons (Li et al. 2020). Moreover, fungal strains of clinical origin assimilate alkylbenzenes. *Exophiala mesophila* isolated from a patient with chronic sinusitis exhibited positive growth on toluene as the sole carbon and energy source similar to *Cladophialophora immunda* which was isolated from a contaminated soil (Blasi et al. 2016). The white‐rot fungus *P. chrysosporium* can degrade an extremely diverse range of persistent/or toxic environmental pollutants. In turn, polychlorinated biphenyls, which are one of the more persistent organopollutants, can be degraded by non-ligninolytic enzymes of pathogenic fungi such as *F. solani*, *P. chrysogenum* and *Scedosporium apiospermum* (Tigini et al. 2009). Finally, fungi produce biosurfactants that have high efficiency in removing toxic elements. Also, yeast species have been utilized as biosurfactants to successfully remove HMs such as Fe, Zn and Pb (Igiri et al. 2018). However, such a phenomenon has yet to be documented in the filamentous representatives.

### HMs as biocontrol agents in fungal disease management

In order to limit the spread of crop diseases different types of fungicides are used, including fungicides containing HMs. While the global use of HM-based fungicides contributes to the dispersal of the HMs throughout different environmental compartments, their antifungal potential is well-documented (Vashistha and Chaundhary 2019). The most common HM-based pesticides are Cu-based. Plant pathogens controlled by Cu-containing fungicides include representatives of oomycetes, *e.g. Plasmopara viticola*, *P. infestans*, as well as fungi such as *A. solani* and *Elsinoë ampelina* (Pérez-Rodríguez et al. 2013; Keiblinger et al. 2018; Battiston et al. 2019). The toxicity results from the ability of Cu to precipitate proteins and cause coagulation of the cytoplasm (Milinović and Đurović, 2007). Many Cu-containing fungicides are prepared using copper hydroxide as the active ingredient and may contain as much as 50% Cu metallic equivalent (Poh et al. 2009). The group of HM-based pesticides may also contain Mn and Zn as the main components. They are widely used to control diseases caused by *Phytophthora* (Luo et al. 2020) and *Peronospora* (Herath Mudiyanselage et al. 2019). The most common Mn/Zn fungicides may contain up to 16% Mn and 2% Zn metallic element equivalent. Some antifungal products available contain even all three elements, *e.g*. ManKocide® with 30% Cu, 3% Mn and 0.4% Zn (Poh et al. 2009). It is worth emphasizing that Zn is also used to control human fungal diseases. Zn-based antifungal compounds such as zinc pyrithione are often administered to treat fungal dandruff caused by *Malassezia* spp., and their toxicity results from increasing the pathogen cellular zinc uptake (Robinson et al. 2021).

There is abundant evidence that classic HM-based fungicides have a long-term effect on soil fauna and flora. The negative effects of their accumulation in soil may occur even at low HM concentrations (Keiblinger et al. 2018). In view of the above, finding safer and eco-friendly alternatives for controlling both fungal and fungal-like diseases needs to be a priority. Nanotechnology as an emerging research domain can be particularly useful in this respect (Sun et al. 2018; Ali et al. 2022). Interestingly, different types of nanoparticles such as zinc oxide nanoparticles (ZnO-NPs) are reported to effectively inhibit pathogenic bacteria, yeasts and filamentous fungi. A wide range of antifungal effects of ZnO-NPs were observed against *C. albicans*, *Trichophyton*, *Microsporum canis*, *mentagrophytes*, *A. flavus*, *Sclerotinia homoeocarpa* and *F. oxysporum* (Sun et al. 2018).

## CONCLUSIONS

**Fig. 3:**
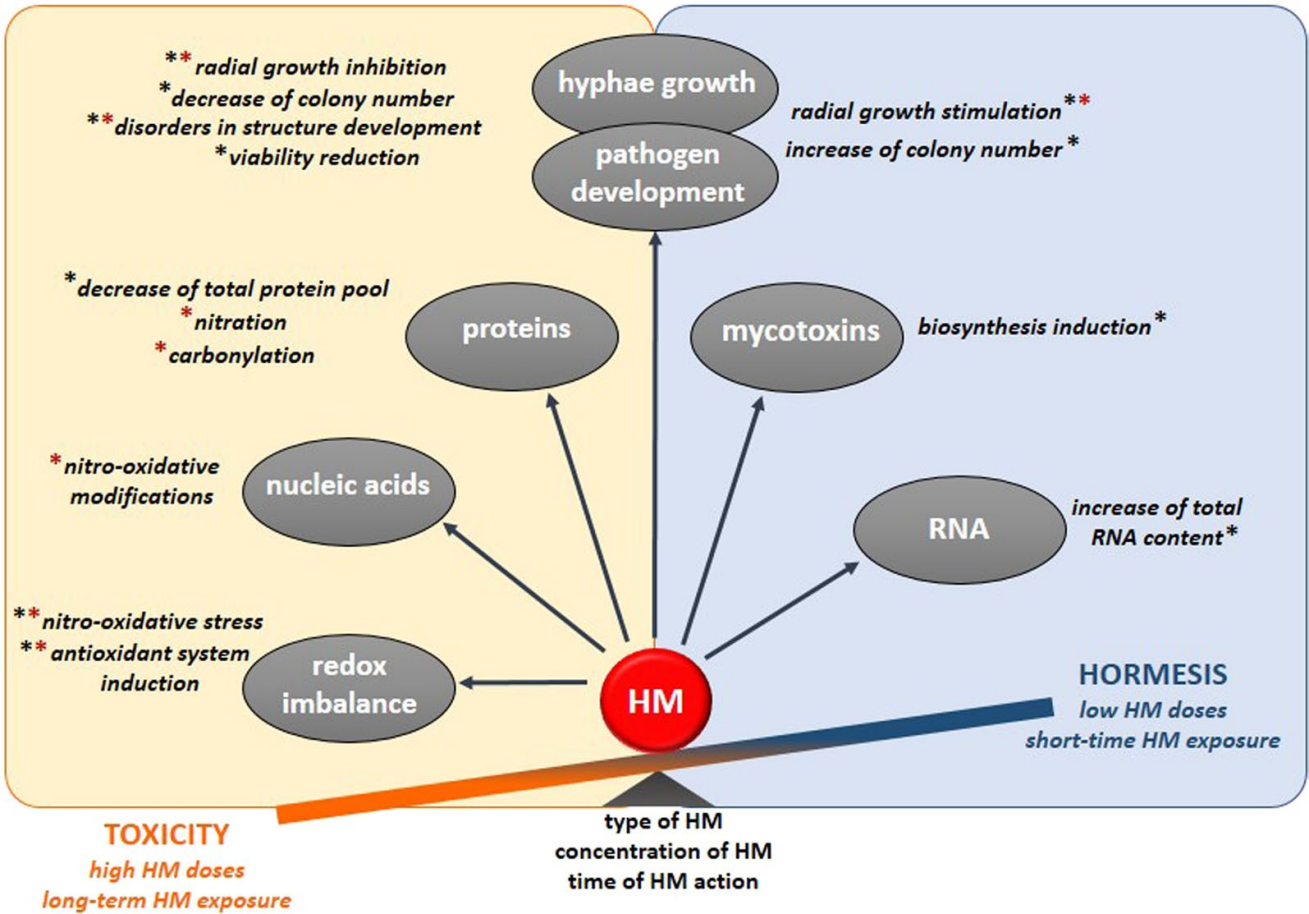
A model summarizing the dual effects of pathogen exposure to HMs. Depending on the HM, its dose and exposure time, a toxic or hormesis-like effects can be observed. Black asterisks indicate effects documented in fungi; red asterisks indicate effects documented in oomycetes

Effects of HMs on filamentous eukaryotic pathogens are multifaceted. Although HMs adversely target hyphal growth, morphology, viability and physiology including nitro-oxidative stress, relatively low HM doses may benefit the pathogen. It was experimentally verified that selected HMs concentrations positively affected growth and some developmental events such as sporulation, resembling hormetic stimulation (Fig. 3). In addition, HM-induced mycotoxin biosynthesis and transcript reprogramming might enhance infection capabilities of microorganisms. For pathogens hormesis may therefore play an important role in their adaptation to a variety of host microenvironments that exhibit different sets of chemical challenges.

The fact that HM-contaminated environment can affect pathogen capability to infect host organisms is of particular importance in regard to economically important pathogens and thus have an impact on agriculture, horticulture and global human health. HMs stress is able to elevate an antioxidant response in fungi and oomycetes at both transcript and enzyme activity levels. Therefore, it can be speculated that pathogenic microorganisms growing in polluted environments are primed to oxidative conditions and thereby can cope effectively with the host’s oxidative defence once the pathogen is recognized. For instance, chronic independent exposure of Bryophytes to HMs such as Cd and Cu adapts them to polluted environments (Boquete et al. 2021). Thus, previous stress history could lead to imprints on long-term effects on the organism’s cellular structure, redox adjustment, bioactive compound levels, composition of mineral and epigenetic modifications. This can then lead to quick and effective response to the second stress event (Floryszak-Wieczorek et al. 2012; Chmielowska-Bąk and Deckert 2021).

Based on the information presented and discussed in this review, the future research focus needs to address the following questions: (1) Do HMs promote or limit pathogenicity and/or virulence and (2) what is the nature of the observed phenomenon? (3) What is the transcriptional response to the hormetic action of HMs? (4) Considering the huge biotechnological potential of fungal microorganisms—should research focus on those pathogenic fungi that have the potential for large scale bioremediation processes? There is a need to characterize the chemistry and environmental fates of the metabolites produced during fungal bioremediation. Finally, HMs are transferred from contaminated soil or water to animal and human organisms via the food chain, thus affecting their natural microbiota. A similar effect may be caused by uncontrolled dietary supplementation with commercial products containing essential elements such as Zn, Fe or Mn. (5) Future research should therefore focus on the identification of the effect of HMs against human and animal microbiota. The growing interest in such issues should help in broadening our knowledge on HM contamination of the microbial environment.

## Data Availability

Not applicable.

## Abbreviations

ABC: ATP-binding cassette
Ag: Silver
As: Arsenic
Ba: Barium
CAT: Catalase
Cd: Cadmium
CDF: Cation diffusion facilitators
Cmt: Cu-detoxifying metallothionein
Co: Cobalt
Cr: Chromium
Cu: Copper
Fe: Iron
Hg: Mercury
HM: Heavy metal
Li: Lithium
LiP: Lignin peroxidase
MATE: Multidrug and toxin extrusion proteins
Mg: Magnesium
Mn: Manganese
Mo: Molybdenum
MnP: Manganese peroxidase
MTs: Metallothionein’s
Ni: Nickiel
PAHs: Polycyclic aromatic hydrocarbons
Pb: Lead
PCs: Phytochelatin’s
PcaA: PIB-type cation ATPase
POX: Peroxidase
RNS: Reactive nitrogen species
R OS: Reactive oxygen species
SOD: Superoxide dismutase
Sr: Strontium
Th: Thorium
U: Uranium
V: Vanadium
Zn: Zinc
ZnO-NPs: Zinc oxide nanoparticles
